# Post traumatic deafness: a pictorial review of CT and MRI findings

**DOI:** 10.1007/s13244-016-0490-9

**Published:** 2016-04-16

**Authors:** Olivier Maillot, Arnaud Attyé, Eric Boyer, Olivier Heck, Adrian Kastler, Sylvie Grand, Sébastien Schmerber, Alexandre Krainik

**Affiliations:** Department of Neuroradiology and MRI, Grenoble University Hospital - SFR RMN Neurosciences, Grenoble, France; University of Grenoble Alpes, IRMaGe, F-38000 Grenoble, France; Department of Otolaryngology-Head and Neck Surgery, University Hospital of Grenoble, Grenoble, France

**Keywords:** Ear ossicles, Trauma, CT scan, Magnetic resonance imaging, Temporal bone deafness

## Abstract

**Abstract:**

Hearing loss is a common functional disorder after trauma, and radiologists should be aware of the ossicular, labyrinthine or brain lesions that may be responsible. After a trauma, use of a systematic approach to explore the main functional components of auditory pathways is essential. Conductive hearing loss is caused by the disruption of the conductive chain, which may be due to ossicular luxation or fracture. This pictorial review firstly describes the normal 2-D and 3-D anatomy of the ossicular chain, including the incudo-malleolar and incudo-stapedial joints. The role of 3-D CT in the post-traumatic evaluation of injury to the temporal bone is then evaluated. In the case of sensorineural hearing loss, CT can detect pneumolabyrinth and signs of perilymphatic fistulae but fails to detect subtle lesions within the inner ear, such as labyrinthine haemorrhage or localized brain axonal damage along central auditory pathways. The role that MRI with 3-D-FLAIR acquisition plays in the detection of inner ear haemorrhage and post-traumatic lesions of the brain parenchyma that may lead to auditory agnosia is also discussed.

***Key Points*:**

• *The most common middle ear injuries are incudo-malleolar and incudo-stapedial joint luxation.*

• *In patients with SNHL, CT can detect pneumolabyrinth or perilymphatic fistula*

• *3-D-FLAIR MRI appears the best sequence to highlight labyrinthine haemorrhage*

• *Axonal damage and brain hematoma may lead to deafness*

## Introduction

### Post-traumatic conductive hearing loss

Post-traumatic conductive hearing loss (CHL) may be due to ossicular chain disruption. Should hearing loss occur after a trauma, symptom chronology must be assessed and audiometric information obtained. In the first few days following injury, hearing may be difficult to evaluate, especially in the event of hemotympanum, which may lead to a transient CHL. Nevertheless, temporal bone trauma is frequently associated with brain and cervical spine injuries [1] that sometimes require surgical management. Six months after a temporal bone trauma, persistent CHL occurs in 50 % of patients [2].

Computed Tomography (CT) allows radiologists to examine the complex anatomy of the temporal bone with submillimeter resolution and is the first modality of choice. Indeed, it is capable of revealing a broad spectrum of ossicular lesions that may not be apparent on the basis of clinical findings alone. Virtual otoscopy with 3-D reconstructions of CT images can provide a different view on ossicular chain anomalies in traumatic conditions [3].

The ossicles range in size from 40 μm to 4 mm yet small damage to any one or just slight alterations in their position can have a large impact on hearing. Radiologists therefore require a strong knowledge base of the normal 2-D and 3-D anatomy of the temporal bone and should possess the ability to interpret CT scans of this complex region. As a useful tool, a high-resolution scan mode with an iterative reconstruction algorithm was found to improve the quality of the temporal bone CT image [4].

### Post-traumatic sensorineural hearing loss

Post-traumatic sensorineural hearing loss (SNHL) potentially involves inner ear lesions such as labyrinthine haemorrhage or perilymphatic fistulae.

The use of unenhanced T1-weighted imaging to detect haemorrhage in the cochlea or vestibule, which appears as high signal intensity in the inner ear, has been assessed in patients with post-traumatic SNHL [5]. Recently, fluid attenuated inversion recovery (FLAIR) imaging appeared useful in the assessment of labyrinthine haemorrhage [6].

Importantly, SNHL may occur in the absence of a temporal bone fracture [5, 7]. One potential diagnosis could be trauma to the membranous labyrinth [8]. In addition, an abnormal communication between the middle and inner ear, known as perilymph fistula, could also cause SNHL via either a leakage of perilymph into the middle ear or pneumolabyrinth in the inner ear. These fistulas most often occur in patients with temporal bone fracture traversing the round or oval window; however, they can also occur with no visible fracture [9, 10]. CT can reveal pneumolabyrinth in the cochlea, the vestibule, or both. Other cases require magnetic resonance imaging (MRI) to detect perilymph inside the middle ear [11, 12] that may be difficult to distinguish from hemotympanum. Finally, the central auditory pathways involve the cochlear nerve, brainstem and the thalamus, the latter of which is connected to the auditory cortex through the auditory radiation. Damage to any one of these sections of the auditory pathways could be held responsible for SNHL and, accounting for the brainstem decussation and depending on the location of the damage, may cause hearing loss on the contralateral side.

## CT and MRI in post traumatic deafness

Images referred to in this article and that were obtained in our institution, involved the following procedures. The CT acquisitions were performed using a 40-section spiral CT scanner (Philips 40; Philips Healthcare®) with the following parameters: 0.5 mm collimation, 0.55 mm section thickness, 140 kV, 300 mAs, a 90 mm field of view and a 1024 × 1024 matrix. The initial data sets were then reconstructed at 0.2 mm intervals. Three-dimensional volume-rendering CT images were generated from the original 2-D data using Philips Intellispace Portal®. A neuroradiology resident or a postprocessing technologist obtained all reformatted images. The application of different soft-tissue and bone algorithms to the 3-D reformation provided a multiprojectional display of the ossicular chain including the joints.

MRI explorations were carried out on a 3T Philips Achieva® TX MR scanner equipped with a 32-channel SENSE receive head coil using a 3-D-FLAIR sequence without injection of contrast media. The FLAIR sequence was performed using the following parameters: TR: 8000 ms, TE: 316 ms, TI: 2400 ms, 0.8 mm isotropic voxel size for acquisition, and 0.4 mm for reconstructions, the SENSE parallel imaging technique with an acceleration factor of 2.5, 2 excitations, and a scan time of 8′40″. A heavily T2-weighted sequence (DRIVE imaging) focused on the inner auditory canal was also performed to assess the cranial nerves.

3-D-FLAIR and DRIVE exploration was added to our standard protocol in patients with trauma, which included susceptibility-weighted imaging and 2D-FLAIR imaging with whole brain coverage.

## External and middle ear

### Normal ear anatomy imaged using 2-D and 3-D CT acquisition

Exhaustiveness and thoroughness are critical in the analysis of post-traumatic temporal bone CT images to ensure that no information from external ear or ossicular structures that could be pathological is overlooked. For this, use of a checklist may be beneficial (Table 1). The precise analysis of tympanic plate morphology is recommended, as a fracture extending through this structure is sufficient to explain otorrhagia. In addition, this lesion is often associated with ossicular fracture or disruption [13].

**Table 1:** Temporal bone CT checklist in a post-traumatic condition

Tympanic plate fracture	Yes or no
Fracture direction	Longitudinal or transverse
Fracture extension to the labyrinth	Yes or no
Liquid in the middle ear	Yes or no
Ossicle luxation	Incudo-malleolar, incudo-stapedial or stapedio-vestibular
Ossicle dislocation	Incus, stapes or incudo-malleolar bloc
Ossicle fracture	Malleus, incus or stapes
Pneumolabyrinth	Yes or No
Facial Nerve Channel	Involved or not

**Fig. 1:**
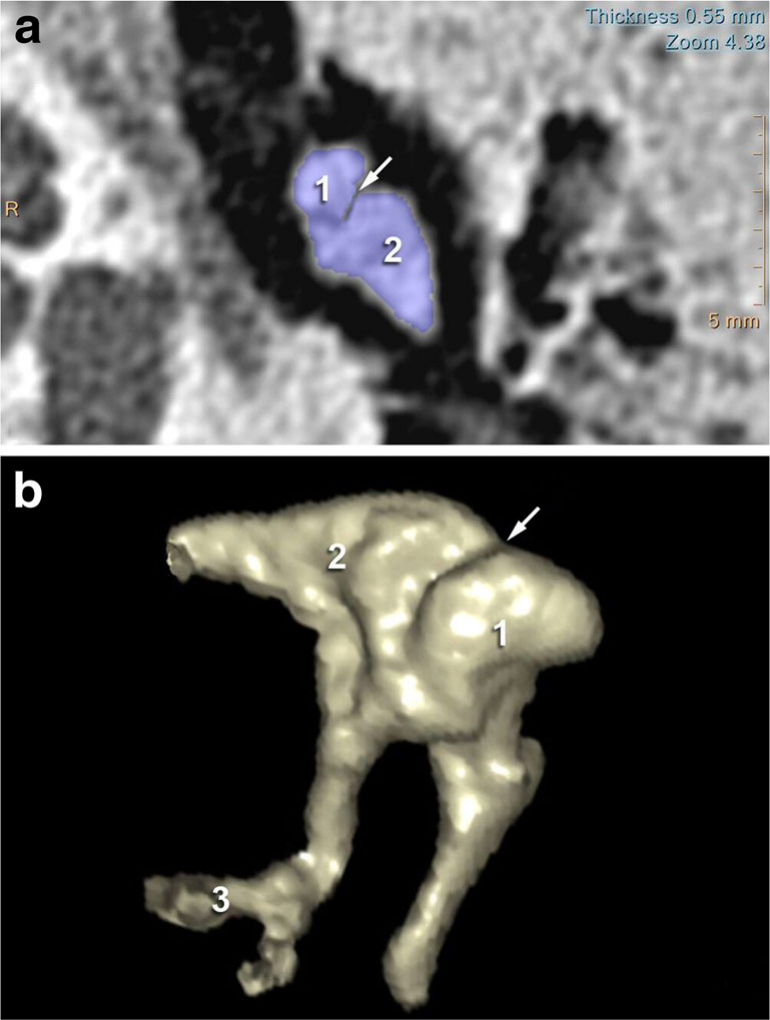
2-D CT images in the axial plane showing a normal incudo-malleolar joint. The ice-cream cone including the body of the incus (2) and the head of the malleus (1) 3-D CT reveals that malleus (1) and incus (2) are in close contact in the healthy middle ear

**Fig. 2:**
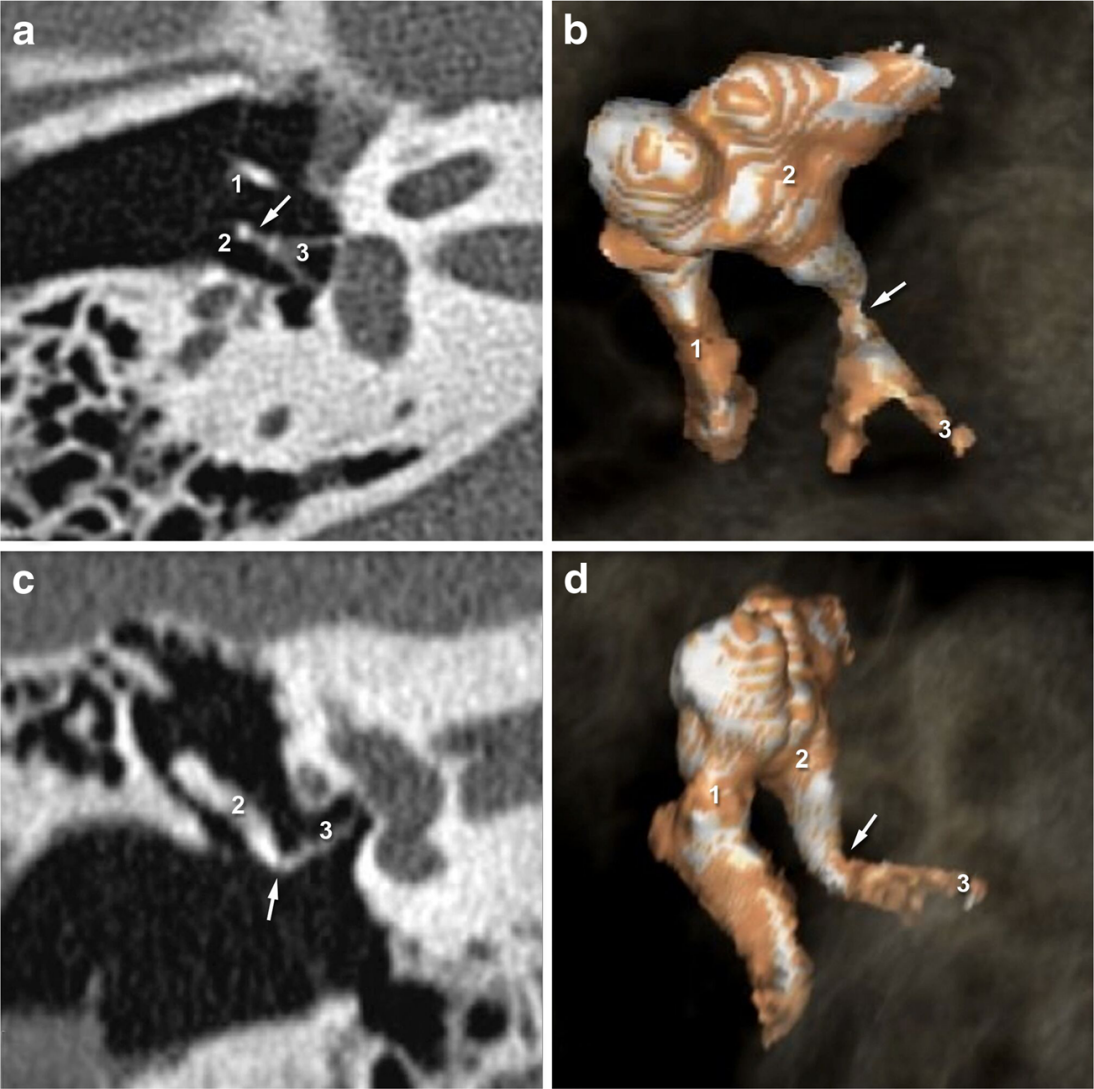
2-D CT images in the axial and oblique planes showing a normal incudo-stapedial joint. The malleus is annotated (1) The axial and oblique planes (**a** and **c**) show the ossicular « V », comprising the long process of the incus (2) and the stapes (3). 3-D-CT acquisition (**b** and **d**) reveal continuity between the stapes head and the long process of the incus in the healthy middle ear

One of the most complex spaces of the temporal bone is the middle ear cavity. Within this space resides the ossicular chain constituted by three ossicles - the malleus, the incus, and the stapes - linked together by two articulations: the incudo-malleolar joint (Fig. 1) and the incudo-stapedial joint (Fig. 2). The incus is the heavier ossicle, with a body and two processes of differing length. The body articulates with the head of the malleus, and the short process is directed posterolaterally while the thin long process runs inferiorly in parallel to the manubrium of the malleus.

Laterally, the tympanic membrane and the handle of the malleus close the middle ear cavity. The head of the malleus is better observed on the upper axial slices at the incudo-malleolar joint. The lower slices show the lenticular process of the incus articulating with the head of the stapes. This is connected to the footplate via the neck, from which emerge the anterior crus and posterior crus.

Finally, assessment of temporal bone pneumatization is important considering the role played by the mastoid portion of the temporal bone in the absorption and dispersion of kinetic energy during direct lateral trauma to the temporal bone [14].

**Fig. 3:**
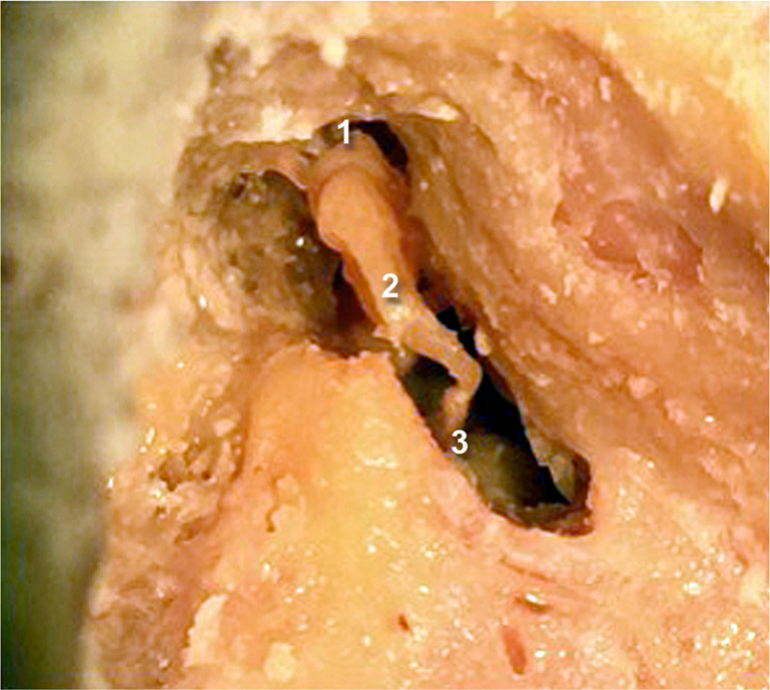
Cadaveric view showing a normal incudo-malleolar joint on the left side and a normal incudo-stapedial joint on the right side. As on the 3-D CT views, the malleus (1) and the incus (2) are in close contact. The incus is also in continuity with the stapes (3)

Please note that cadaveric views (Fig. 3) are provided in this article to better illustrate normal and pathologic ossicular joints.

### Injury imaging using 2D and 3D CT acquisition

The 3D reconstruction of data from temporal bone CT increases the likelihood of identifying traumatically injured structures of the temporal bone due to the ability to rotate in space and study incudo-malleolar and incudo-stapedial joints in different planes.

#### Incudo-stapedial joint injuries

**Fig. 4:**
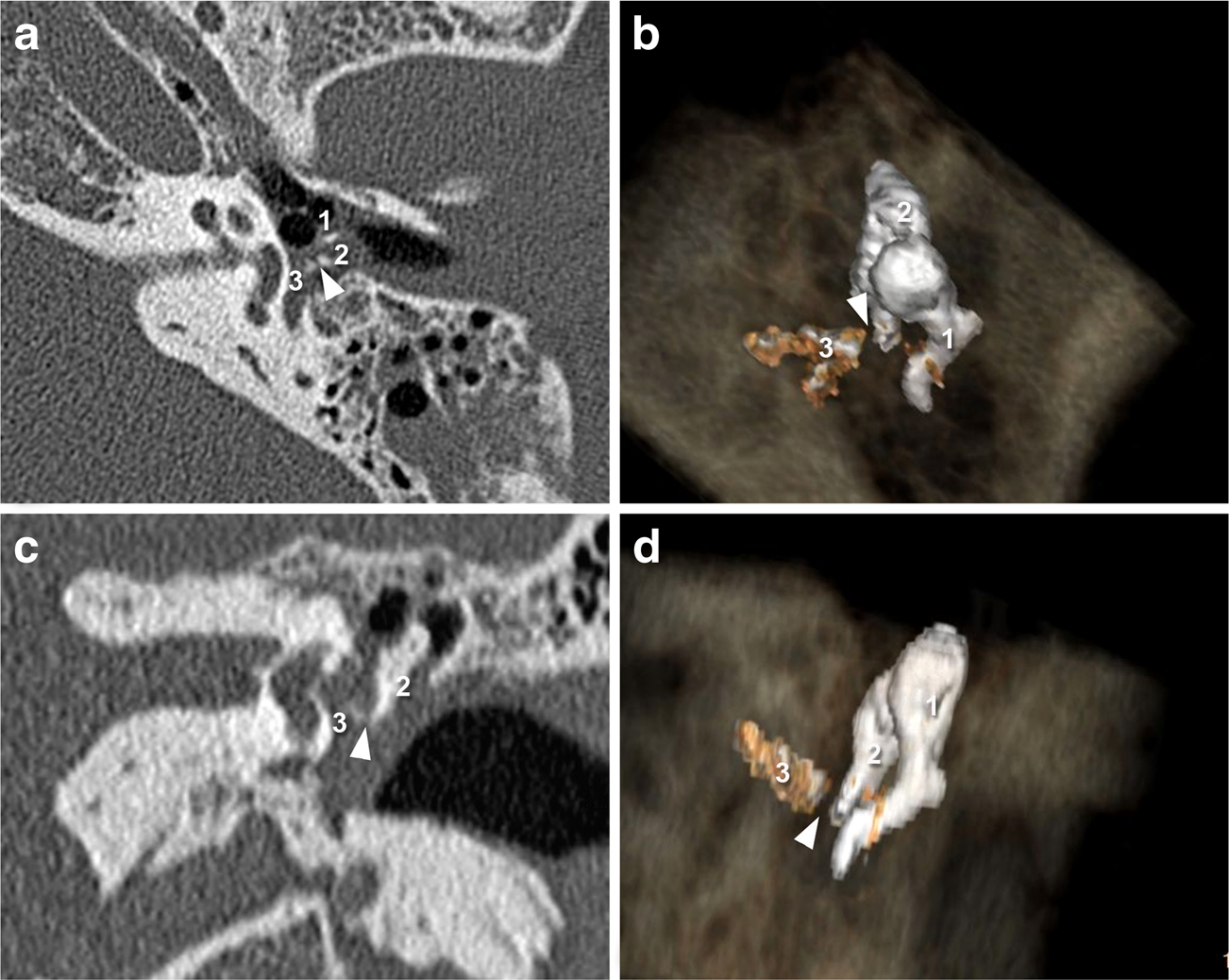
2-D CT images in the axial and oblique planes (**a** and **c**) showing a post-traumatic incudo-stapedial joint luxation. The malleus is annotated (1) The oblique plane shows disruption of the ossicular « V », comprising the stapes (3) and the long process of the incus (2). The 3-D CT images (**b** and **d**) also reveal the “gap” (*white arrowhead*) between the stapes (3) and the incus (2)

The best view of this joint if searching for potential trauma is provided in the oblique plane (perpendicular to the oval window). This plane shows any lack of contact between the lenticular process of the incus and the head of the stapes [15]. In this plane, the ossicular chain usually represents a “V” formed by the long process of the incus and the stapes. This may however be disrupted due to luxation, as revealed by a “gap” between the incus and stapes. 3-D acquisitions are particularly useful in the assessment of this lesion (Fig. 4), even in patients with hemotympanum for whom isolation of the stapes superstructure may be difficult [16].

The incudo-stapedial joint is the most commonly affected by ossicular luxation, as diagnosed upon post-traumatic exploration of the middle ear by a ENT surgeon [17]. By contrast, radiological data traditionally failed to obtain such incudo-stapedial luxation incidence, probably related to the associated hemotympanum.

#### Incudo-malleolar joint injuries

**Fig. 5:**
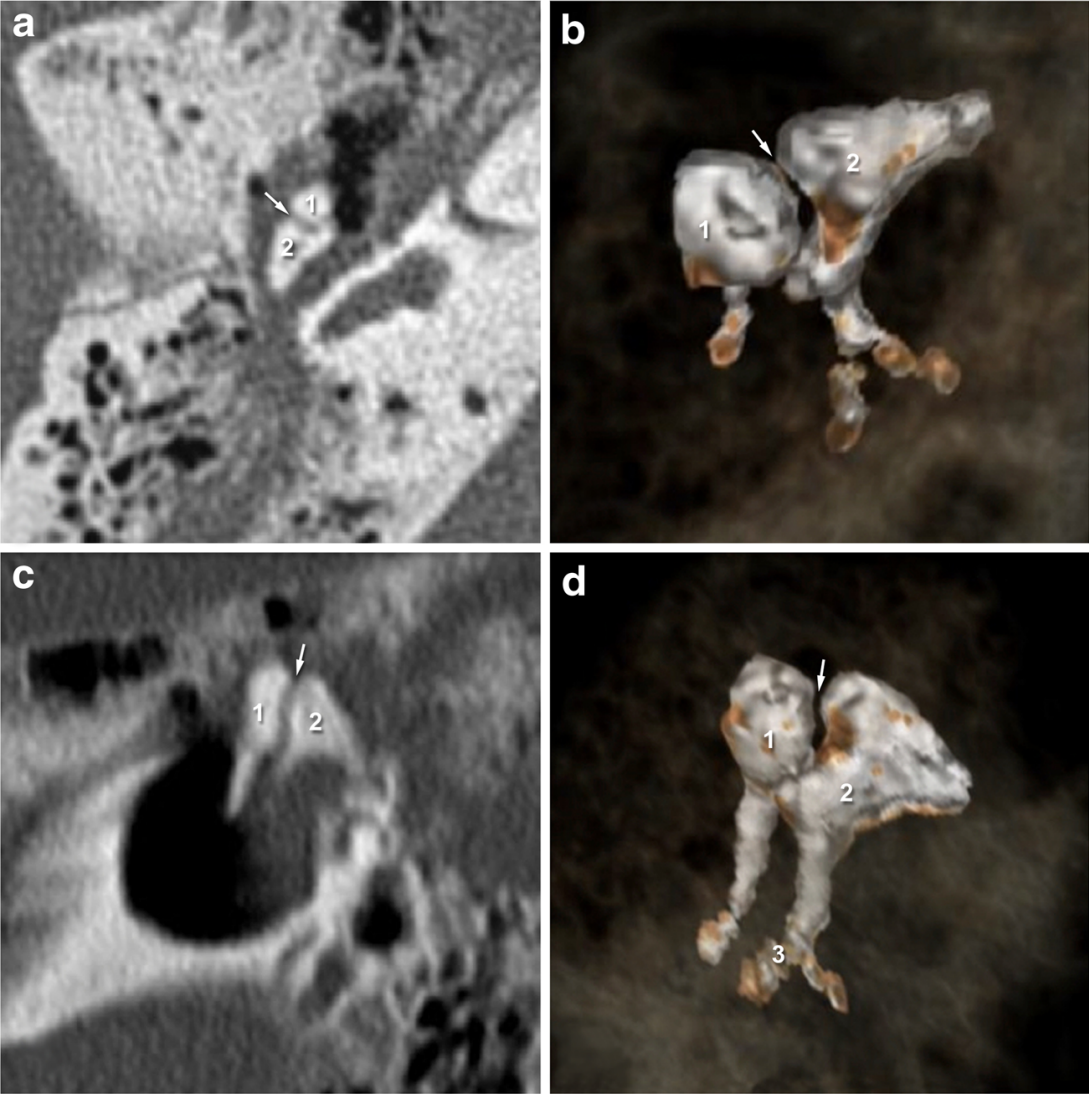
2-D-CT images (**a** and **c**) in the axial and coronal planes showing the incudo-malleolar joint after temporal bone trauma. The incus (2) is no longer in contact with the head of the malleus (1) on 2-D and 3-D-CT acquisition (**b** and **d**)

The incudo-malleolar joint looks like an ice cream cone: the head of the malleus corresponding to the scoop of ice cream and the body and the short process of the incus corresponding to the cone. These structures should be in close contact on the axial plane view. A luxation or subluxation is defined by a lack of osseous continuity that can also be studied on the coronal plane view useful to detect commonly occurring small lesions [15]. As with incudo-stapedial disarticulation, 3-D volume-rendering reconstructions may also help to detect such lesions in the incudo-malleolar joint (Fig. 5).

Besides luxation and subluxation, incudo-malleolar dislocation occurs upon violent trauma associated with a longitudinal T-bone fracture. In such cases, the malleus head and the incus body remain fixed together but the entire bloc is displaced. The incudo-malleolar complex is often displaced inwards.

**Fig. 6:**
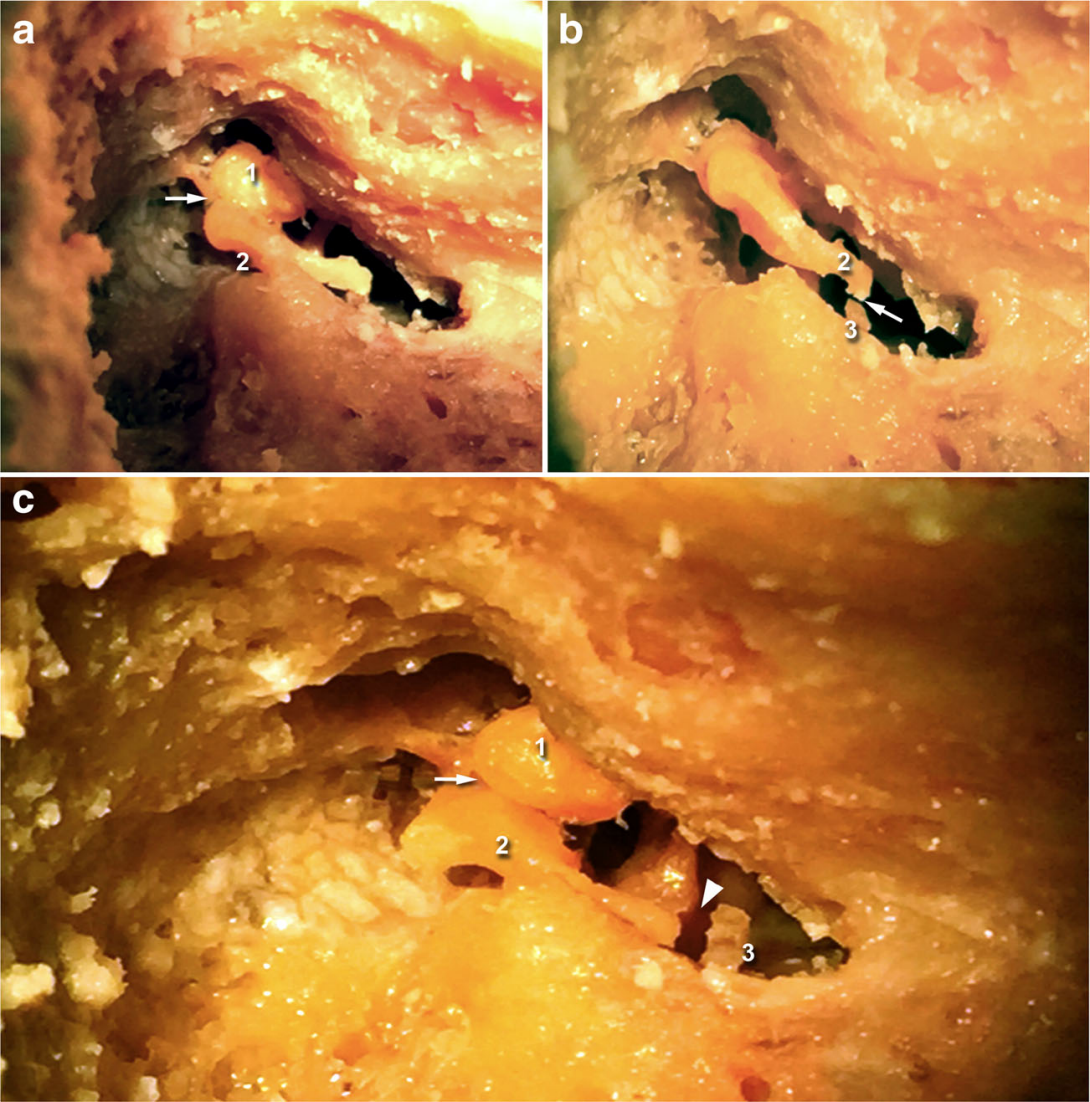
Cadaveric view showing incudo-malleolar luxation (*white arrow*, upper left) and incudo-stapedial luxation (*white arrow*, upper right). On the third image, both joints are dislocated (*white arrow* and *white arrowhead*). The malleus is annotated (1), the incus (2), and the stapes (3)

The incudo-stapedial and incudo-malleolar joint luxations have also been illustrated on cadaveric data (Fig. 6).

#### Incus dislocation

When both incudo-malleolar and incudo-stapedial disarticulations occur, the incus is dislocated. The displacement of the incus is variable in terms of direction and amplitude. In the case of lateral incus dislocation, a “Y” shaped configuration of the incudo-malleolar complex is seen in the coronal view despite the axial views presenting a normal ice cream cone configuration [18].

#### Stapediovestibular luxation and stapes dislocation

**Fig. 7:**
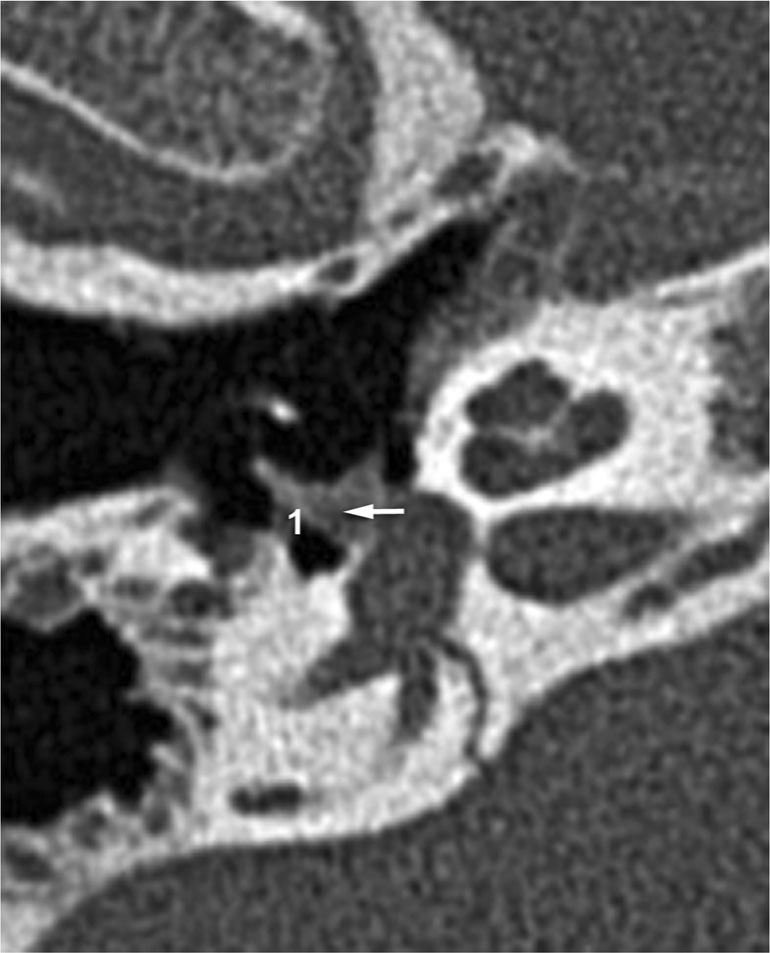
Stapedio-vestibular luxation: CT stapes view highlighting stapedio-vestibular luxation. The perilymphatic liquid leaking through the oval window (1) implies rupture of the annular ligament

Stapediovestibular luxations are rare lesions that are most commonly caused by direct and penetrating trauma to the external ear canal [19, 20], though they have also be associated with incudomalleolar disarticulation and stapes fracture [21]. The footplate can also be dislocated in the tympanic cavity due to a traumatic force tearing the annular ligament via an increase in perilymphatic pressure (external dislocation) [22]. An annular ligament lesion may lead to a perilymphatic fistula (Fig. 7). In such cases, patients present with cochleovestibular symptoms including SNHL, tinnitus, and acute vestibulopathy.

#### Isolated ossicular fractures

Isolated ossicular fractures are less frequent than joint luxation and may involve any one part of the chain.

Malleus fractures usually involve the handle and may be accurately diagnosed using high resolution CT with reformatting of images through the handle [23]. These should be suspected in cases of sudden hearing loss after digital manipulation of the external auditory canal [24].

Fractures of the incus most frequently affect the long process due to its fragility, with those affecting the body or the lenticular process occurring more rarely. The preferential planes for their visualization are axial and coronal with reformatting through the axis of the long process.

Injuries to the stapes may be difficult to diagnose at the early stage due to hemotympanum. More severe high frequency hearing loss has been described in patients with stapes superstructure fractures compared to that occurring in those with incudo-stapedial disarticulation [25]. Fracture of the footplate occurs secondarily to a transverse fracture passing through the oval window, and may cause a perilymphatic fistula with pneumolabyrinth.

## Inner ear and brain

### Normal inner ear anatomy and central auditory pathways on MRI acquired images

Magnetic resonance is the modality of choice when investigating the inner ear after a trauma. The routine protocols include a heavily T2-weighted sequence and a T1-weighted sequence without gadolinium enhancement. More recently, the FLAIR sequence is emerging as a complementary tool to the standard commonly used protocol [6]. Without gadolinium enhancement, it is of interest in the investigation of intra-labyrinthine haemorrhage. By contrast, its use is also expanding to highlight endolymphatic hydrops, requiring first the injection of contrast media before acquiring images 4 hours later [26].

**Fig. 8:**
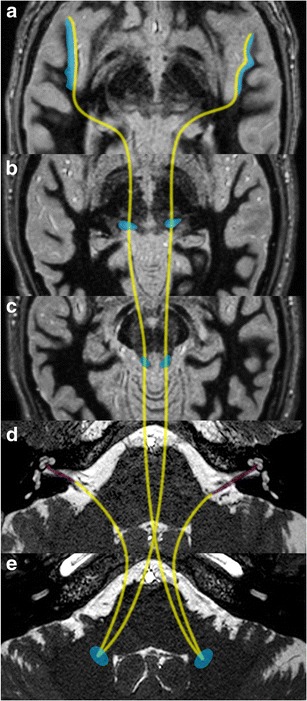
Central auditory pathways: the auditory cortex is located in the superior temporal gyrus (**a**: *blue areas*). Beyond the cochlear nerves (**d**: *coloured in purple*); the central auditory pathways run through the cochlear nuclei, the inferior colliculii (**c**) before they decussate to the contralateral olivary nucleus (**e**), run through the medial geniculate body (**b**) and finally reach the auditory radiations

Faced with a lack of evidence of inner ear post-traumatic disease, the central auditory pathways should be investigated in their entirety. The principal auditory pathway leading to the cerebral cortex passes from the cochlea, via the cochlear nerve, cochlear nuclei, the inferior colliculus, and the medial geniculate body to the contralateral auditory cortex in the temporal lobe (Fig. 8). The various auditory centres in the brainstem not only serve as way stations to the ascending auditory pathways, but are also relays for descending auditory projections [27].

### Post-traumatic inner ear haemorrhage, perilymphatic fistulae and endolymphatic hydrops

**Fig. 9:**
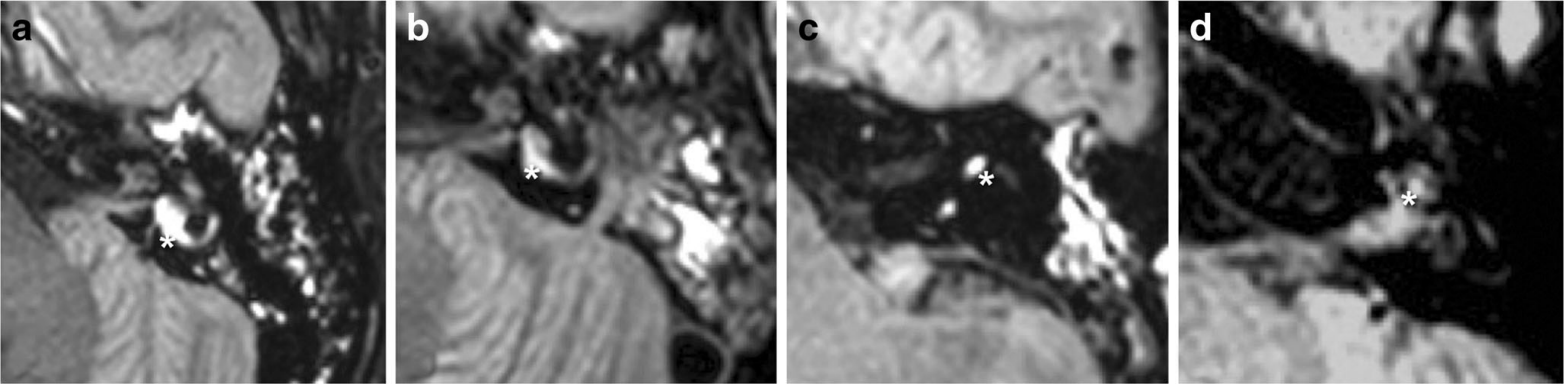
Examples of inner ear haemorrhage: the inner ear haemorrhage (*white star*) appears as a hypersignal on nonenhanced FLAIR acquired images of the vestibule (**a** and **b**), superior semicircular canal (**c**), and the cochlea (**d**). The first and third patient (**a** and **c**) referred with trans-labyrinthine fracture, the second (**b**) with extra-labyrinthine fracture, and the fourth (**d**) with major brain trauma, without temporal bone fracture. All patients presented post-traumatic SNHL, ipsilateral to the haemorrhage

**Fig. 10:**
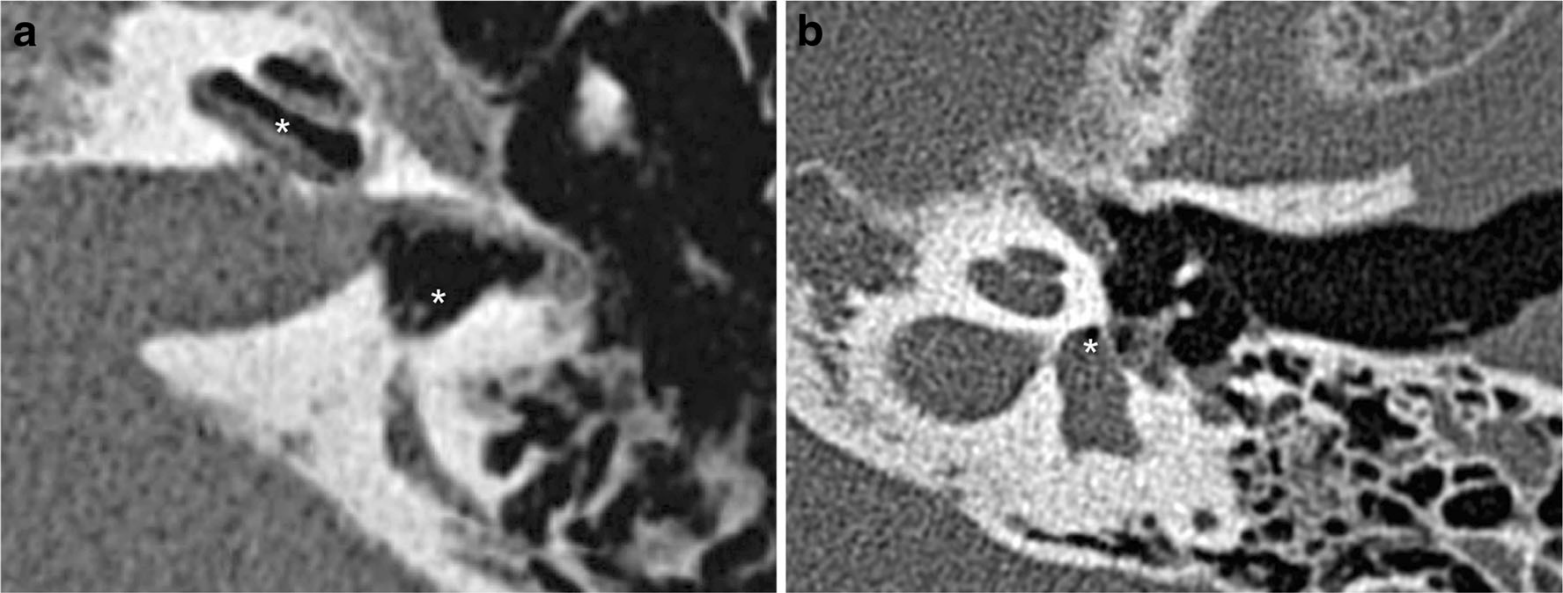
**a** Perilymphatic fistulae: CT axial views showing a perilymphatic fistula, highlighted by a pneumolabyrinth (*white stars*). Perilymphatic liquid has leaked into the middle ear. Massive pneumolabyrinth was seen 1 month after a translabyrinthine fracture, with air in the perilymphatic space (scala vestibuli and the vestibule) **b** Example of pneumolabyrinth without temporal bone fracture (*white star*)

A 3-D approach towards imaging the inner ear provides an essential assessment of the complete set of labyrinthine structures in their entirety. The main symptoms of labyrinthine concussion are hearing loss, tinnitus, and dizziness. High frequencies are more vulnerable to trauma than low frequencies [28]. The diagnosis mainly relies on audiometric testing, which can reveal characteristic tracings reminiscent of acoustic trauma, and MRI. Unenhanced T1-weighted spin echo sequence before gadolinium injection was initially described as being an essential tool for the study of post-traumatic hearing loss with the aim of diagnosing intralabyrinthine methemoglobin [29]. However, 3-D-FLAIR has since been shown as more sensitive than T1-weighted imaging in detecting subtle compositional changes of lymphatic fluid [30]. Non-enhanced FLAIR acquisition may reveal a hypersignal inside the cochlea, vestibule or both (Fig. 10) [7]. One case of isolated semicircular canal haemorrhage was described using FLAIR imaging [31] (also seen in Fig. 9).

**Fig. 11:**
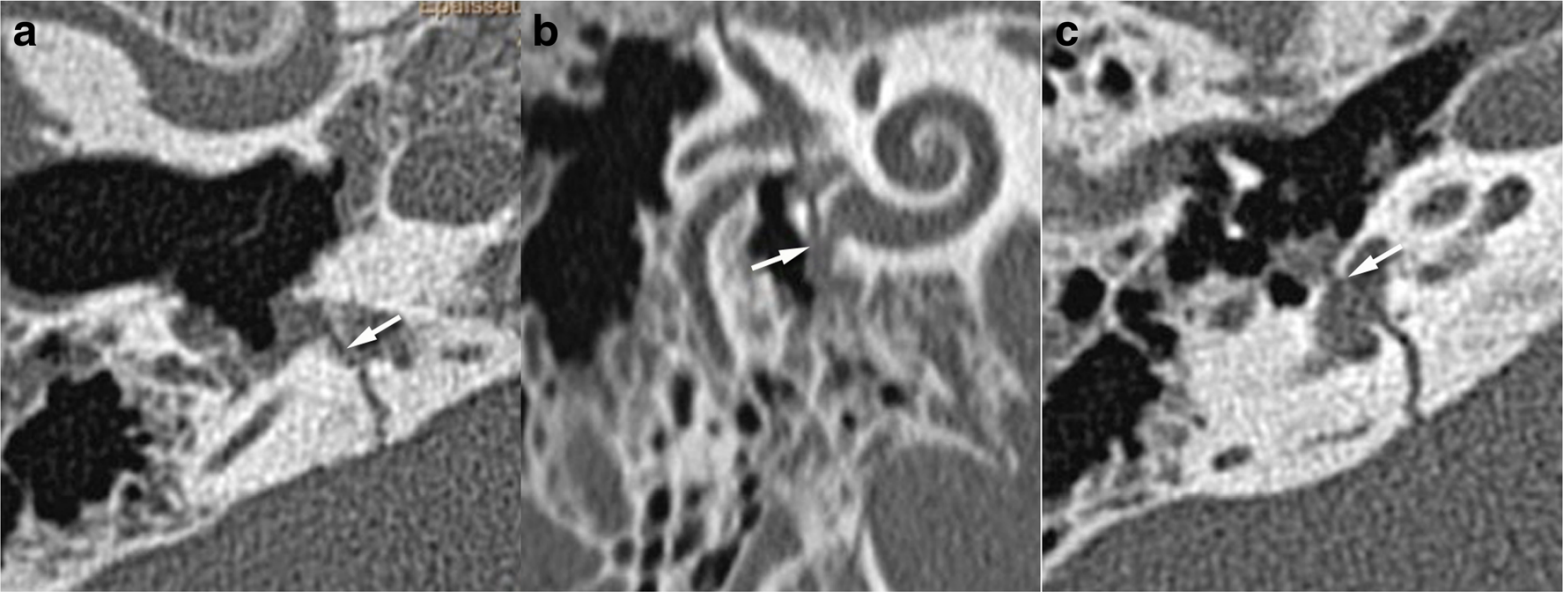
Perilymphatic fistulae: round and oval windows are the two communication routes between the middle ear and inner ear, implying two weakness zones. Axial and oblique CT views showing fractures (*white arrows*) crossing the round (**a**, **b**) and oval (**c**) windows

A perilymphatic fistula is a direct communication between the middle ear and inner ear cavities. This may be seen without associated temporal bone fracture [32] and appears indirectly linked to pneumolabyrinth and pneumocochlea (Fig. 10). There are two zones of weakness between these two cavities: the oval window and the round window. The oval window is affected either by a footplate fracture or by a lesion to the annular ligament, which may be isolated or associated with stapedio-vestibular disarticulations or stapes dislocation (external or internal). When the round window is affected, the fracture line can be seen around the edges of the window. It must be researched in the axial and coronal planes (Fig. 11). A pneumolabyrinth can be seen in the absence of temporal bone fracture [33].

**Fig. 12:**
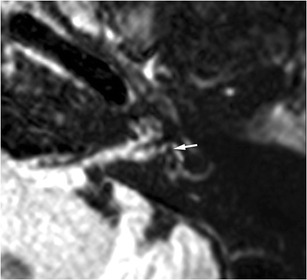
Post-traumatic endolymphatic hydrops: for this patient with extra-labyrinthine fracture and post-traumatic left SNHL, the vestibular hydrops was obvious with an endolymphatic vestibular area (*white arrow*), encompassing up to 50 % of the whole vestibular surface. However, the role of trauma as a direct cause of the endolymphatic hydrops remains unclear

Besides perilymphatic fistulae, endolymphatic hydrops has been highlighted in patients with Meniere’s disease or Recurrent peripheral vestibulopathy using MRI with single-dose injection of contrast media and delayed acquisition [26, 34]. Post-traumatic hydrops is a true clinical entity based on clinical observations and histopathologic study [35]. Thus, in patients with SNHL with neither inner ear haemorrhage nor fistulae, we perform the FLAIR sequence 4 hours after contrast media injection to look for a post-traumatic endolymphatic hydrops (Fig. 12).

### Injury to the central auditory pathways

It is important to distinguish injuries that occur along the central auditory pathways before the decussation into the superior olivary nucleus that may lead to asymmetrical SNHL, from those potentially responsible for associated auditory agnosia [36]. This condition refers to impairments in sound perception and identification despite intact hearing, cognitive functioning, and language abilities [37].

The lesions that may lead to SNHL involve cochlear nerves or post-traumatic brainstem hematoma. Cochlear nerves could be injured by a superficial leptomeningeal hemosiderosis that may be seen after head trauma [38]. The diagnosis of hemosiderosis often relies on MRI with susceptibility-weighted imaging [39].

**Fig. 13:**
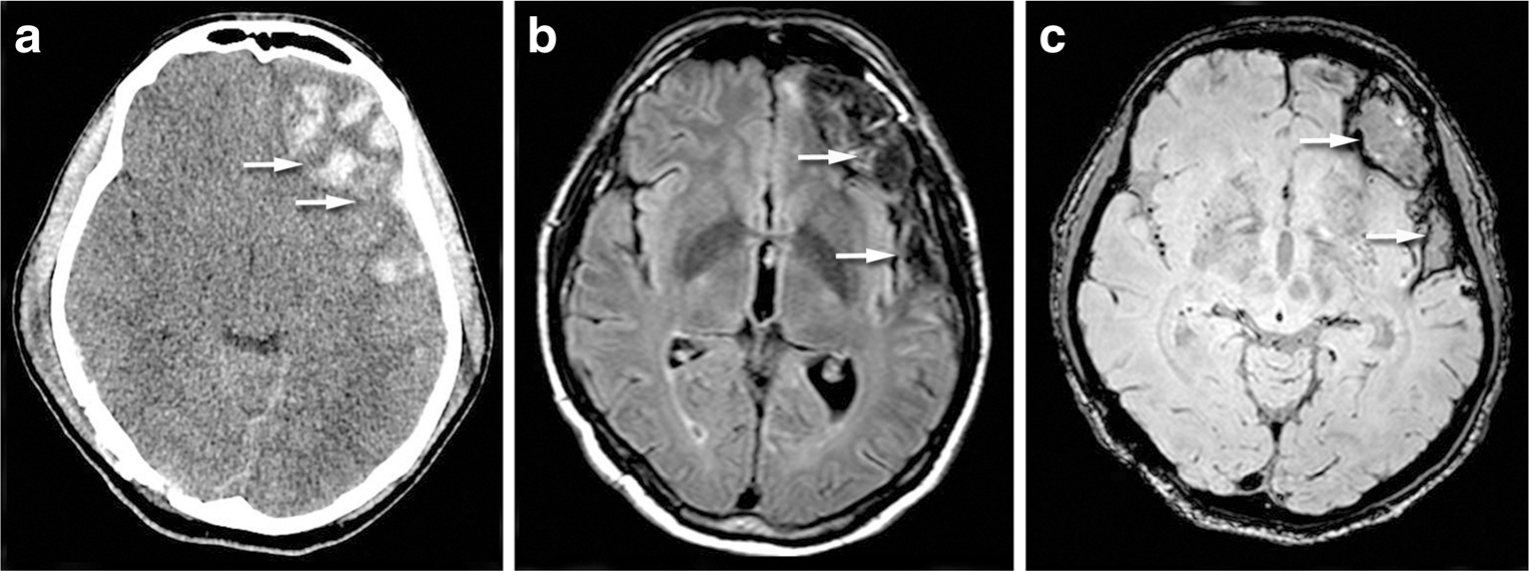
Brain haemorrhage in a patient with contralateral auditory agnosia. CT view (**a**) showing left fronto-temporal hemorrhagic injury. FLAIR sequence (**b**) and susceptibility-weighted imaging (**c**) showing the cortical damage in particular in the superior temporal gyrus and in the basifrontal region (*white arrows*)

Theoretically, isolated injury of the thalamus, auditory radiations, or auditory cortex could alter auditory function. Isolation of auditory radiations requires advanced diffusion techniques, such as MR tractography, while the auditory cortex can be visualized with functional MRI [40, 41]. Post-traumatic cerebral contusion located along the Heschl’s gyrii may be responsible for SNHL (Fig. 13). New MR techniques such as susceptibility-weighted imaging [42] or track-weighted imaging [43] will likely prove useful in the assessment of focal brain lesions after trauma, including mild traumatic brain injury.

## Conclusion

To conclude, the most common middle ear injuries affecting CHL are incudo-malleolar and incudo-stapedial joint luxation.

SNHL may appear after severe brain injury or temporal bone trauma and potentially have major functional consequences. CT can detect pneumolabyrinth or perilymphatic fistula while FLAIR MRI appears the best approach to highlight inner ear bleeding. Finally, the post-traumatic brain injuries that may be responsible for deafness are axonal damage and brain hematoma.
